# Small animal patient preoperative preparation: a review of common antiseptics, comparison studies, and resistance

**DOI:** 10.3389/fvets.2024.1374826

**Published:** 2024-03-28

**Authors:** Alicia K. Nye, Kelley M. Thieman Mankin

**Affiliations:** Department of Small Animal Clinical Sciences, College of Veterinary Medicine and Biomedical Sciences, Texas A&M University, College Station, TX, United States

**Keywords:** antiseptic, antiseptic action, surgery, skin preparation agents, skin preparation techniques

## Abstract

This review aims to describe commonly used antiseptics in veterinary medicine including their mechanism of action, spectrum of activity, potential adverse effects, and application techniques. Additionally, it provides a review of the veterinary literature comparing antiseptics, a discussion of effectiveness and efficacy studies, and the potential for increased resistance to biocides and antimicrobials. This review concludes that appropriate selection and use is necessary to prevent the occurrence of surgical site infections, adverse effects, and potential for increasing resistance to antimicrobials. Continued research is needed to fill gaps in the current knowledge such as optimal preparation procedures for various surgical sites, standardization of efficacy and effectiveness testing, and the clinical impact of decreased susceptibility to chlorhexidine and other antiseptics.

## 1 Introduction

Surgical site infections (SSIs) pose a major challenge in veterinary medicine regarding antibiotic treatment selection, prevention of hospital acquired infections, and zoonotic risks. Studies of veterinary SSIs performed in the past two decades report an incidence of approximately 3–6% for all procedures (1–5) with a range of 1.7% for minimally invasive procedures (6) to 15.8% for certain orthopedic procedures (7). In addition to increased cost, antibiotic use, and patient morbidity associated with SSIs, the proximity of companion animals to humans and potential for zoonotic infections requires optimization of strategies for SSI prevention in veterinary hospitals.

Aseptic preparation of patients involves the use of antiseptics preoperatively in order to prevent wound contamination that may lead to the development of infections. Surgical asepsis is defined as the absence of microorganisms within any type of invasive procedure. However, up to 20% of the resident flora of skin is beyond the reach of antiseptics, therefore aseptic preparation of patients truly involves a reduction of the resident flora and the removal, inhibition, or destruction of transient and pathogenic organisms on the proposed operative site (8).

The origination of surgical asepsis in the 19th century was brought about in response to high postoperative mortality rates following surgical wound infections. In 1865 it was discovered by Joseph Lister, a surgeon known as a pioneer in antiseptic surgery, that application of carbolic acid-soaked dressings decreased the occurrence of infections. Over a decade later, physician and microbiologist Robert Koch was able to link bacteria to wound infections. He performed a series of experiments assessing the efficacy of different antiseptic agents. Through the efforts of many additional surgeons, physicians, and scientists over the following decades, surgical asepsis evolved to include standards in patient, surgeon, instrument, and environmental preparation that define modern surgical practices (9).

Today, antiseptics are chosen based on their effectiveness in killing bacteria and other microorganisms, rapidity of action, safety, ease of use, and persistence of action, among other criteria. The most commonly used antiseptics for surgical preparation of patients include chlorhexidine, iodine compounds, and alcohols (10).

## 2 Chlorhexidine

Chlorhexidine (CHX) is a cationic bisbiguanide disinfectant and antiseptic. Its mechanism of action is rooted in its ability to bind to and disrupt the bacterial cell membrane. The outermost layer of the bacterial cell has a net negative charge due to components of the cell wall including teichoic acid and polysaccharides of Gram-positive bacteria, lipopolysaccharide of Gram-negative bacteria, and the deeper cell membrane. The cationic biguanide groupings within the molecule bind strongly to negatively charged sites, particularly proteins and the head groups of acidic phospholipids on cell membranes, causing displacement of divalent cations that aid in stabilization of the wall and membrane. The distance between CHX’s phospholipid binding sites are roughly equivalent to the distance between phospholipid heads in a tightly packed monolayer, making it capable of forming a bridge between two adjacent membrane phospholipids. At low concentrations, CHX causes reduced membrane fluidity and affects the cell membrane and associated enzymes osmoregulatory and metabolic capabilities. This leads to cellular leakage of potassium ions and protons, and inhibition of respiration and solute transport within the bacteria. As concentrations increase, the membrane transforms from a fluid to a liquid-crystalline state, leading to a loss of structural integrity, leakage of cellular materials, and bacterial cell death. High concentrations cause coagulation of intracellular constituents and congealment of the cytoplasm, allowing CHX to disrupt and kill bacterial cells without cell lysis (11, 12).

CHX’s mechanism of action allows for a broad spectrum of antimicrobial activity. CHX is most active against Gram-positive bacteria, but also has activity against Gram-negative bacteria, anaerobes, fungi and some enveloped viruses. It is ineffective against bacterial spores and is mycobacteristatic (10, 11, 13). It is rapidly taken up by bacteria and yeasts, with the maximum effect occurring within 20 s (10, 11). Its ability to bind to protein in the stratum corneum of the epidermis allows for persistence of action (8) which has been shown to have a cumulative effect with repeated use (14). The residual activity of CHX has been shown to last at least 24 h on skin and this is extended to 48 h when combined with alcohol in a tincture (a solution made with alcohol as its solvent) (15). Interestingly, a study by Rutter et al. evaluated CHX’s residual activity by application of *S. aureus* to participant’s hands through dry-contact contamination. The residual activity was only appreciated when participant’s hands were re-wetted after contamination, indicating that it only produces a residual kill when in solution. However, the authors did not evaluate residual activity in areas of *de-facto* moisture (e.g., gloved hands, moist body regions) or investigate the suppression of resident microflora growth (16). Anionic thickeners or emulsifiers, as found in certain moisturizing products and hand sanitizers, have been found to inactivate the persistence of action of CHX (17–19).

CHX is available as gluconate or diacetate salt formulations. Scrub preparations typically contain detergents and alcohol, while aqueous formulations are free of these substances. Inactive ingredients vary depending on the proprietary formula used by the manufacturer. CHX gluconate scrub is the most common agent used on intact skin for patient preparation in veterinary clinics (20). While CHX diacetate (Nolvasan Solution, Fort Dodge Animal Health, Fort Dodge, IA) has been used on dog skin (10), it is not labeled for use on skin, but instead is labeled as a cleaner for animal premises. In 2010, a 2% CHX gluconate aqueous solution came on the market as Vet One (MWI Animal Health, Boise, ID), labeled for use on intact or damaged skin of horses and dogs. Regarding storage of CHX, open containers of 4% CHX gluconate exposed to artificial and natural bacterial contamination over a course of 6 months have proven to resist bacterial growth (21), however dilute preparations (<2%) are susceptible to bacterial contamination and should not be pre-mixed and stored (11, 22).

Currently, no standard exists for veterinary patient preparation prior to surgery. Several CHX formulations are not labeled for this specific use or for use on animals. Protocols for patient preparation therefore vary between veterinary clinics and staff responsible for this preoperative step are sometimes unaware of the concentration being used as well as recommended contact times (20). The clinical significance of this is not known, since it has been shown that concentrations of scrub ranging from 1.0 to 4.0% are effective in adequately reducing bacterial load after 3 min of contact time (20). Regarding application, no difference has been found between mechanical (continuous scrubbing during predetermined contact time) and non-mechanical (quick application of scrub followed by allotment of same contact time) aseptic preparation (23) or technique for mechanical application (linear scrubbing vs. concentric circles) (24). Preparation protocols often include alternating alcohol and CHX or using alcohol to remove excess scrub on the skin after a preparation with CHX. Osuna et al. found that a 4% CHX gluconate scrub alternated with saline or 70% isopropyl alcohol performed similarly in their mean bacterial reduction and percentage of samples with negative cultures. However, the study found that CHX gluconate removed with alcohol resulted in more positive bacterial cultures postoperatively, suggesting that the addition of alcohol may affect the residual activity of CHX. The authors questioned the significance of this finding, recommending that a study evaluating the incidence of surgical site infections after these preparation methods be performed (25). While this particular study has not been performed on veterinary patients to date, it is important to note that current recommendations favor the use of antiseptics combined with alcohol for intraoperative skin preparation, based on studies in the human literature (26).

Human studies vary as far as benefits of preoperative bathing or showering using skin antiseptics to prevent SSI. Recent reviews of the human literature show no clear evidence that preoperative showering or bathing with chlorhexidine reduces SSIs when compared to other wash products (27). In 2017, a systematic review with meta-analysis evaluating preoperative bathing of the surgical site with CHX for SSI prevention was performed (28). The metanalysis gathered data on over 10,000 patients and a significant reduction in SSI was not found when comparing patients subjected to preoperative bathing with CHX to patients in the placebo group. Still, some human studies report lower rates of SSI when CHX is used to cleanse the surgical site the night before surgery (29, 30). In 2017, the Centers for Disease Control and Prevention published guidelines for the prevention of SSI in human surgery and included the recommendation to perform a full body shower or bath with soap or antiseptic agent at least the night before surgery (26). In humans, the skin surface concentration of CHX is maximized with a standardized protocol including allowing the CHX to stay on the skin for at least 1 min prior to rinsing (31). One study has been performed in dogs to assess bacterial counts in limbs washed with CHX gluconate the night before sampling compared to limbs that were not washed. In that study, the treated limb did have a lower bacteria count, however, no difference was detected in skin bacterial counts following the full routine preoperative CHX scrub and alcohol disinfection (32).

For open skin wounds, a concentration of 0.05% aqueous CHX is recommended. This is the minimum concentration shown to be bactericidal against *S. aureus*, no matter if physiologic saline, balanced electrolyte solution, or sterile water is used for dilution (33, 34). CHX diacetate at concentrations higher than 0.006% has been shown to be cytotoxic to canine embryonic fibroblasts *in vitro*, however a 0.05% concentration does not affect wound healing or wound contraction *in vivo* (35). CHX diacetate is known to precipitate in some solutions, such as Lactated Ringer’s Solution, but this does not impact its effectiveness (34). At low concentrations (<0.16%), CHX’s bactericidal effect may be reduced in the presence of organic matter, such as serum, possibly due to protein binding (36). Therefore, CHX may not be as effective when low concentrations are used for cleaning wounds with large amounts of organic contamination. At CHX concentrations above 1.6%, organic matter does not impact its ability to disinfect (37), but does lead to increased cytotoxicity.

Overall, CHX causes few adverse reactions. CHX is known to be ototoxic at concentrations at or above 0.5%, abolishing all vestibular and auditory potentials (38, 39). However, CHX does not cause ototoxic effects at concentrations of 0.2%, making the recommended concentration of 0.05% safe for flushing the external ear canal of dogs without an intact tympanum (40, 41). Cats, however, may display transient ototoxicity and middle ear mucosal injury even at this low concentration (42). For preparation of the oral cavity, a 0.2% gluconate solution is recommended (43). Importantly, CHX is known to cause severe keratitis and corneal ulceration with potential permanent corneal damage at high concentrations or when combined with a detergent (44–46). At low concentrations, the degree of corneal toxicity differs between the gluconate and diacetate formulations. A concentration of 0.05% CHX gluconate does not cause gross or microscopic changes to the cornea and it is used routinely for preparation of the conjunctiva prior to ophthalmic surgery and as a contact lens preservative (47, 48). However, CHX diacetate at this same concentration causes corneal toxicity at the gross and microscopic levels (47). Due to these well-known ophthalmologic and otologic adverse reactions, previous reports have concluded that CHX should not be used in preparation of skin on the head (46). Intraoperatively, CHX should not be instilled into the peritoneum, bladder, or synovial joints due to the potential for chemical peritonitis, erosive cystitis, and synovitis (49–51). Systemic toxicity has not been reported with CHX; its binding to the skin prevents it from being readily absorbed and there is also negligible absorption through the alimentary tract (8, 49, 52). Reports of hypersensitivity in humans range in degree of severity from urticaria to life-threatening anaphylaxis (53). Most cases are associated with the application of CHX to mucous membranes and areas of damaged epidermis. However, incidence of hypersensitivity may be underestimated due to poor recognition of this adverse reaction (54).

## 3 Iodine compounds

Iodine compounds are halogen-releasing disinfectants and antiseptics. They readily penetrate the microorganism’s cell wall, target and oxidize proteins, nucleotides, and fatty acids in the cytoplasm and cell membrane, resulting in cell death. Free, or molecular iodine (I2), has a broad spectrum of antimicrobial activity effective against bacteria, fungi, viruses, and spores (11, 55, 56). In more than 150 years of use, no bacterial resistance to iodophors has been reported experimentally or anecdotally (57).

Tinctures containing alcohol and unstable aqueous solutions were historically used as antiseptics but fell out of favor due to irritation, toxicity, and excessive staining (10, 11). Iodophors, such as polyvinylpyrrolidone-iodine (povidone-iodine; PI) or poloxamer-iodine, contain iodine complexed with a polymer. Though less active against spores and fungi than tinctures, less free iodine in these water-soluble complexes results in them being less irritating and more stable, and therefore these preparations are most commonly used today (58). Additionally, these complexes enhance the antimicrobial efficiency of iodine due to their affinity for the cell membrane, improve its wetting properties and assist in its penetration into organic material (55).

Iodophors comprise an equilibrium of complexed and free iodine which acts as a reservoir for the release of the halogen as it is used up (55). The amount of free iodine, versus “available iodine,” is responsible for a solution’s microbial activity. For example, a 10% PI solution contains 1% available iodine and 1 mg/L (1 ppm or 0.0001%) free iodine, meeting recommendations that antiseptics contain 1–2 mg/L of free iodine (55, 59). The quantity of free iodine within a solution can be illustrated with a bell-shaped curve. An approximately 0.7% solution strength contains the highest content of free iodine, and this content is reduced as the solution concentration moves away from this value in either direction (55). While this bell-shaped curve applies to the various manufactured formulations, the exact amount of free iodine and bactericidal activity may vary between products (60).

The enhanced bactericidal activity of dilute solutions, compared to full-strength 10% solutions, has been shown *in vitro* on various bacterial species (61). Various organic materials have been found to neutralize the effects of iodophors, which is thought to be due to iodine forming complexes and undergoing chemical reactions, such as reduction to iodide. Neutralization of iodophors occurs especially in the presence of whole blood, given hemoglobin’s ability to bind a large amount of iodine (55), but also occurs in the face of purulent material and fat homogenate (62). This finding may actually support the use of higher concentrations of PI when organic matter is present, as dilute solutions have a decreased reservoir of iodine, and therefore may be more readily neutralized (63). Iodophors display moderate persistence which is greatly extended up to 48 h when combined with alcohol (25, 64, 65). Regarding storage, contamination of commercially available iodophors has occurred and spouts of PI jugs have cultured positive for *S. aureus*, illustrating these multi-use containers are a potential source of contamination (66, 67).

PI is a commonly used iodophor in veterinary medicine for patient preparation (10). Formulations may contain alcohol or detergents and vary in concentrations up to 10%. All formulations are recommended for mucous membranes and external use only, however recent human guidelines recommend irrigation of the deep and subcutaneous layers of incisional wounds with dilute iodophor solutions intraoperatively to reduce the incidence of surgical site infections (26, 68). This recommendation was recently evaluated in total hip arthroplasty in veterinary patients. Postoperative SSI rates were evaluated in arthroplasties performed with or without a pre-closure lavage of 0.035% PI solution and was found to be efficacious in lowering infection rates, safe, and cost effective (69). Surgical scrub formulations contain detergents for scrubbing (55). For preparation of skin on the head, a 10% solution free of alcohol and detergent, which could disrupt the tear film of the cornea, is currently considered the safest (46). For preparation of the cornea and conjunctiva, a 0.2% solution of PI was found to be equally bactericidal as a 1 or 5% solution (70). However, a concentration of 5% is recommended for prophylaxis with intraocular surgery, as it is safe to use and may be more effective in patients with high bacterial load (71). Like CHX, a tincture of 0.7% iodine povacrylex in 74% isopropyl alcohol (DuraPrep) is more effective than 10% PI in reducing positive skin cultures immediately after application to human skin (72). Ioban, a PI impregnated adhesive incise drape, is a product used to reduce the risk of wound contamination and offer continuous antimicrobial activity according to the manufacturer’s website. To assess its effectiveness in veterinary patients that have a denser haircoat and more surface debris, Osuna et al. compared a PI scrub and alcohol prep to preparation with alcohol and application of the antimicrobial drape. The authors found the drape preparation to be inferior to PI scrub in reducing bacterial load on the skin (73). Additionally, a potential to increase infection rates was found by a human literature meta-analysis assessing the adhesive drapes. This finding may be explained by an increase in moisture under the adhesive drape encouraging bacteria residing in hair follicles (those out of reach of antiseptics) to migrate to the surface and multiply (73–75). The value of incise drapes, with or without antiseptic impregnation, is therefore questionable in veterinary medicine, given the high incidence of non-adherence (67). DuraPrep solution enhances adhesion between surgical drapes and the prepared skin surface, but this application has not been evaluated on the skin of veterinary patients (64).

To determine an effective yet safe concentration for wound irrigation, Sanchez et al. evaluated the cytotoxicity of various concentrations of PI to *in vitro S. aureus* and canine embryonic fibroblasts as well as their effect on wound healing. The group performing this experiment found that a concentration of at least 1.0% PI is required for complete killing of *S. aureus in vitro*, and while this concentration was found to be cytotoxic to fibroblasts, it had no effect on wound contraction and healing *in vivo* (33, 35). Thus, a 1.0% concentration of PI solution is the current recommendation for wound treatment.

Adverse effects are related to topical irritation and increased systemic levels of iodine. Acute contact dermatitis, erythema, wheals, edema, papules, and weeping of serum may occur in nearly half of dogs prepped with PI (25, 76). Dilute solutions appropriate for application to mucous membranes also have the ability to produce tissue reactions, such as in preparation of the canine preputial cavity (77). PI administered through any route can result in systemic absorption. This appears to be enhanced when in contact with denuded skin, mucosal or serosal surfaces, or large areas of intact skin (57). Systemic absorption may lead to metabolic abnormalities and abnormal thyroid function. Due to these potential derangements and the clearance of iodine by the kidneys, PI wound preparation is not recommended in cases of burns, thyroid disease, or renal disease (55, 57, 78).

## 4 Alcohols

Alcohols, such as ethyl alcohol and isopropyl alcohol, act as disinfectants and antiseptics. Their exact mechanism of action is not known, but they are believed to cause membrane damage and protein denaturation, resulting in interference with metabolism and cell lysis (10, 11). They have a broad spectrum of activity against Gram-positive and Gram-negative bacteria, viruses, and fungi, but are not sporicidal. Water is necessary to increase alcohol’s microbicidal efficiency, with formulations of 60–90% alcohol being most effective (59). While the concentration of the product is more important than the type of alcohol, isopropyl alcohol has greater bactericidal action than ethanol (10, 59).

Alcohol provides the fastest and greatest reduction of bacteria on the skin, but it has no residual activity and therefore its efficacy is limited to its contact time before drying (10, 11, 59). A 70% concentration of ethanol can eliminate 90% of skin flora with a 2-min application time. However, due to the quick drying nature of this agent and its common use as a means of removing residual antiseptic during preparation, this bacterial reduction is typically not achieved. For example, a single wipe with ethanol only reduces cutaneous bacteria by 75% (8). Efficacy is improved by the addition of emollients that decrease alcohol’s evaporation time, such as in waterless hand sanitizers, and the addition of other antiseptics such as CHX and iodophors (8, 11). In fact, alcohol potentiates the action of these added antiseptics, resulting in greater microbicidal activity and increasing their residual action (59, 72, 79, 80). Studies evaluating the efficacy of alcohol in the presence of organic matter are limited, but alcohol appears to be effective in the presence of small amounts of blood; however, it is not a good cleansing agent and therefore dirt should be removed prior to application (59, 81).

Alcohol may be used as part of a combination product with CHX or iodophors or used as part of an initial preparation to remove excess antiseptic. Alcohol should be allowed to dry completely for full effectiveness as well as to decrease irritation and flammability, which is a risk when using electrosurgery. The duration recommended for an alcohol-based solution to dry is a minimum of 3 min after application to hairless skin; application or pooling of solution in haired skin should be avoided as it can take up to 1 h to dry. In pediatric veterinary patients, an alcohol rinse used during preparation does not significantly decrease rectal temperatures when compared to a rinse with water (82). Additional adverse effects of alcohol applied to the skin are minimal, but absorption through intact skin has been demonstrated and was associated with infant toxicities in the 1950s (59, 83). However, even with a combination of dermal absorption and inhalation of vapors, central nervous system depression is unlikely without prolonged (several hours) exposure to alcohol (84). When compared to ethanol, isopropyl alcohol is slightly more toxic and causes more vasodilation leading to increased bleeding at puncture sites and incisions (8, 85). Due to its cytotoxicity, alcohol is not indicated for application to wounds or mucous membranes (10).

## 5 Comparison of antiseptics

CHX, PI and alcohol have different mechanisms of action, risks and benefits which are summarized in Table 1. Several systematic reviews and meta-analyses have compared CHX to PI in the human literature and found CHX to be superior to PI in both effectiveness outcomes evaluated (86–89). Studies have reported that CHX is superior to PI in reducing SSIs, especially in clean-contaminated surgeries, with two of the reviews finding a moderate quality of evidence for a 30–36% reduction (86, 89). Two different reviews could not conclude which antiseptic was more effective (90, 91). Kamel et al. stated that overall conclusions could not be made, especially regarding the active ingredient of the antiseptics used, as they may be mixed with either saline or alcohol (90). Dumville et al. concluded that due to the lack of clear evidence, practitioners should base their decisions on other characteristics such as cost and potential side effects (91). The most recent meta-analysis by Chen et al. found no difference in adverse events when comparing the CHX and PI (88). Multiple reviews cite several limitations, including variation in the antiseptic concentration and formulation, application technique, patient population, study design, and in some, not taking into account the role of alcohol in their results.

**Table 1:** Mechanisms of action, recommended concentrations, toxicities and limitations of chlorhexidine, iodine compounds and alcohols when used as preoperative preparation.

Antiseptic	Activity	Recommended concentrations for non-cutaneous preparation (alcohol and detergent-free formulations)	Toxicity	Limitations and resistance
Chlorhexidine (CHX)	Broad spectrum: most active against Gram +, also activity against Gram −, anaerobes, fungi and some enveloped viruses Binds protein in skin resulting in persistence of action for 24 h +	Open skin wounds: 0.05% Ear canal: 0.05% (dogs) Oral cavity: 0.2% gluconate Ocular: 0.05% gluconate	Caution if used on head Ototoxicity at >0.5% concentration in dogs (causes transient ototoxity and mucosal injury in cats at this concentration) Severe keratitis and corneal ulceration at high concentrations of gluconate or when combined with detergent Not to be used in peritoneum, bladder or synovial joints	Dilute preparations (<2%) are susceptible to contamination and should not be premixed and stored Performs better when combined with alcohol Neutralized by organic material at <0.16% concentration Resistance leading to decreased susceptibility
Iodine compounds (PI)	Broad spectrum: bacteria, fungi, viruses, and spores Some persistence of action	Open skin wounds: 1.0% Skin of head: 10% Ocular: 5%	Can cause skin irritation and excessive staining Systemic absorption can cause metabolic abnormalities including abnormal thyroid function Not recommended for patients with burns, thyroid or renal disease	Multi use containers can be contaminated with bacteria Performs better when combined with alcohol Neutralized by organic material No reported resistance
Alcohols	Broad spectrum: bacteria, fungi, and viruses No persistence of action Potentiates action of CHX and PI and increases their persistence of action	Not recommended as non-cutaneous preparation	Central nervous system depression possible with prolonged exposure to vapors or absorption through intact skin	Quick evaporation decreases contact time and efficacy Not a good cleansing agent Bacteria are less likely to develop resistance

Due to differences between the skin of humans and that of veterinary species, species-specific findings are likely more appropriate, however, veterinary literature on the subject is rare. Recently, a systematic review and meta-analysis of the veterinary literature comparing CHX and PI was published (92). The authors found no difference in the incidence of postoperative SSIs or skin bacterial colonization between protocols using the two antiseptics. However, the limited number of studies and variable risk of bias within the studies accounted for an overall low quality of evidence (92).

Very few prospective studies in the small animal veterinary literature compare CHX to PI at their recommended concentrations using similar protocols for preparation. Amber et al. investigated the effectiveness of CHX and PI aqueous solutions at various dilute concentrations in reducing bacterial load in artificially contaminated open wounds. Investigators found CHX-treated wounds had a significantly decreased bacterial load 48 h later when compared to PI-treated wounds (93). Comparison of dilute CHX and PI for reduction of bacterial load in the canine prepuce was performed by Neihaus et al.; it was found that preparation with CHX resulted in a significant decrease in the proportion of positive cultures post flush when compared with PI (77). Conversely, Nye et al. evaluated dilute preparations of CHX and PI in the initial preparation of the external car of dogs prior to ear canal surgery and failed to find a difference in reduction of bacterial load between the two antiseptics (94). Phillips et al. also failed to find a difference between the antiseptics when comparing the incidence of SSI in sites prepared with either CHX scrub followed by an aqueous solution, or PI scrub followed by an aqueous solution (95). Sanchez et al., evaluated the effectiveness of daily irrigation with dilute concentrations of CHX and PI in preventing wound contamination; the authors found that neither antiseptic was fully effective in preventing contamination, but CHX resulted in significantly fewer contaminated wounds throughout the 14-day experiment (35).

Several animal studies have been performed to evaluate unmatched antiseptic concentrations and combinations. Belo et al. found no difference between the effectiveness of an aqueous 7.5% PI solution and an alcoholic 2% CHX solution in reducing skin bacteria in dogs (96). When comparing 0.7% PI in 74% isopropyl alcohol (DuraPrep) to 4% CHX gluconate scrub, Gibson et al. found no difference in antiseptic efficacy or the incidence of skin reactions (97). No difference was found by Osuna et al. in the incidence of negative cultures up to 1 h after antiseptic preparation when comparing 4% CHX gluconate alternated with alcohol and 10% PI scrub alternated with alcohol and application of 10% PI solution, but dogs in the PI group exhibited significantly more skin reactions (76). Swaim et al. investigated the bacterial load of bandaged canine paws immediately after preparation and 24 h after preparation with 7.5% PI scrub and 10% PI solution versus 2% CHX scrub and solution. Results indicated similar effectiveness and residual activity of the two antiseptics, and no further decrease in bacterial load after initial application (98). More recently, Asimus et al. found no difference in bacterial load after dog skin was prepped with either 4% CHX scrub alternated with saline rinse or a mild shampoo rinsed with saline followed by an alcohol-based gel. The alcohol gel protocol was found to be faster with the added theoretical benefit of preventing the emergence of bacterial resistance to CHX (99). These inconsistent results represent the larger conflicting body of human and veterinary literature, and while CHX appears to be favored in more than one meta-analysis, several variables, including the contribution of alcohol, are unclear (100).

Both CHX and PI tend to perform better when combined with alcohol, as found in *in vivo* and human studies. *In vivo*, Adams et al. compared the efficacy of 2% CHX gluconate in 70% isopropyl alcohol (ChloraPrep) to 2% CHX solution in reducing bacterial load. ChloraPrep resulted in a greater reduction of bacteria, especially when a biofilm carrier test was performed with and without the addition of 10% human serum (79). When comparing ChloraPrep and Duraprep in human colorectal surgeries, which are inherently high risk for developing infections, Kaoutzanis et al. found no difference in the incidence of SSIs (101). A meta-analysis by Peel et al. comparing alcohol-based surgical site skin preparation (either combined products or the addition of an alcohol step) found a reduction in SSIs with the use of CHX and alcohol versus iodophor and alcohol. However, due to the low number of studies and potential for bias, it was unclear if this superiority applied to all surgical procedure groups (102). Combined antiseptic-alcohol preparations are convenient due to their one-step application process, but it is worth mentioning that the area must still be decontaminated with a non-medicated soap, dry, and residue-free prior to application for maximum effectiveness according to manufacturer instructions.

## 6 Bacterial resistance to antiseptics

No standardized methods exist for testing susceptibility, and no consensus exists on the definition of antiseptic resistance. Various terms are used to define the degrees of bacterial susceptibility. Bacteria described as insusceptible are considered intrinsically resistant to the antiseptic. Phenotypic tolerance is defined as bacterial survival in the presence of an antiseptic after a phenotypic change or transient condition. Antiseptic tolerance implies that the bacteria strain is inhibited, but not killed, by the antiseptic. Finally, resistance may be defined as a bacterial strain that can survive exposure to an antiseptic at a concentration that kills the rest of the bacterial population (13).

When evaluating for resistance, susceptibility may be determined by several methods including minimum inhibitory concentration (MIC), minimum bactericidal concentration (MBC), or time-kill studies. Results are highly dependent on the method of testing and there are no agreed upon breakpoint values (13). MIC methods determine the lowest possible concentration required to inhibit growth of the organism and therefore evaluate antiseptic concentrations well below what is used clinically. Because antiseptics are aimed toward bacterial killing versus inhibition, and MIC tests below in-use concentrations, MBC or time-kill studies may be more appropriate when testing for resistance. MBC methods measure the lowest possible concentration required to kill the organism and allow for comparison of resistant and susceptible strains. However, like MIC, it relates to concentrations attainable by body fluids, which is not relevant to the topical administration of antiseptics (13). Time-kill studies measure the growth of bacteria exposed to an antiseptic in broth for different lengths of time and antimicrobial activity is determined by the rate of reduction in the number of microorganisms; bactericidal is typically defined as a log_10_ reduction factor of 5 (13). Testing of antiseptics is affected by numerous variables and it has been suggested that the method for testing susceptibility should mimic “in-use” conditions in regards to the culture media and culture characteristics, however most of the evidence of resistance comes from laboratory based experiments (103).

Mechanisms of bacterial resistance to antiseptics, like antimicrobials, are either a natural property of the bacteria (intrinsic) or acquired. Intrinsic properties typically consist of an impermeability of the organism’s outer layers to the antiseptic and less commonly, degradation of the antiseptic by the constitutively synthesized enzymes. Bacterial spores display intrinsic resistance to antiseptics due to their cortex and inner and outer spore coats. The complex cell wall of mycobacteria provides intermediate resistance to many antiseptics. In general, Gram-negative bacteria display more intrinsic resistance than Gram-positives due to the composition of their outer cell membrane, notably with *Pseudomonas aeruginosa*, *Proteus* spp., *Burkholderia cepacia*, and *Providencia stuartii*. In Gram-positive bacteria, intrinsic resistance may be due to reduced diffusion through the extracellular glycocalyx and mucopolysaccharides, such as the slime layer produced by mucoid strains of *Staphylococcus aureus* (11). Phenotypic resistance can be achieved through intrinsic properties like biofilm formation and render a colony resistant to various antiseptics. Biofilms consist of a conglomerate of sessile organisms organized within an exopolysaccharide polymer that display reduced sensitivity to antiseptics and are of particular concern with nosocomial and implant-associated infections. Biofilm-mediated resistance may be due to decreased permeability to the antiseptic, chemical interaction between the antiseptic and the biofilm, modulation of the microenvironment, production of degradative enzymes, or genetic exchange between cells within the biofilm. Bacteria that exhibit biofilm formation include *Staphylococcus (S. aureus and S. pseudintermedius), Pseudomonas aeruginosa, Acinetobacter, Actinobacillus, and Klebsiella* (104).

Acquired resistance can occur through the acquisition of mobile genetic elements (plasmids and transposons), mutation, or induction. Efflux pumps may be acquired through mobile genetic elements and aid in resistance by decreasing the intracellular concentration of the antiseptic. *Staphylococcus aureus* plasmids contain the *qacA-G* gene families that encode proton-dependent export proteins that impart low-level resistance to multiple antiseptics. Efflux pumps are also responsible for reduced antiseptic efficacy in *Pseudomonas aeruginosa* and *Escherichia coli* (103, 105). Induction or mutation caused by stress may lead to acquired resistance to antiseptics. This may be responsible for changes in cell permeability, transcription of resistance genes, or other mechanisms of resistance. Mutation or selection may be responsible for the decreased antiseptic susceptibility of Gram-negative bacteria isolated from hospital environments (11).

In addition to the potential for decreased susceptibility to commonly used antiseptics, there is concern that acquired antiseptic resistance may confer mechanisms that result in antibiotic resistance. Overlap of resistance between antimicrobials is in part due to these organisms sharing common resistance mechanisms, such as biofilm formation, induction of resistance genes, changes in the cell envelope, and nonspecific efflux pumps. However, laboratory experiments have identified several methods that result in resistance to antibiotics following antiseptic exposure. Cross-resistance involves selection for genes that encode resistance to both antiseptics and antibiotics. For example, the export proteins associated with the *qacAB* gene family in *S. aureus* plasmids display significant homology to other energy-dependent transporters, such as tetracycline exporters (11). Co-resistance is the selection of clones or mobile elements that carry genetically linked resistance genes affecting antiseptic and antibiotic susceptibility, such as *S. aureus* plasmids that carry resistance genes for various antibiotics, heavy metals, and antiseptics. Finally, biocide exposure may lead to indirect selection of a bacterial sub-population that displays reduced susceptibility to both antimicrobial agents (103).

CHX resistance is of particular importance given its widespread usage and multiple reports of decreased efficacy against problematic organisms such as methicillin-resistant *S. aureus* (13). Bacterial spores, mycobacteria, and several Gram-negative bacteria such as *Pseudomonas, Proteus*, and *Providencia* spp. display variable resistance to CHX due to intrinsic factors. More importantly, however, are the mechanisms of acquired resistance that have led to multiple reports of decreased susceptibility to CHX. In the Gram-positive *S. aureus*, the efflux pump encoded by the *qacA* gene is commonly associated with reduced susceptibility to CHX and may be found on multiple plasmids that also carry resistance genes to other antimicrobials. This gene has a significantly higher prevalence in isolates from nurses when compared to the general population, suggesting that repeated exposure to CHX may result in selection for these genes (13). It is important to note that the presence of the *qac* genes alone does not indicate CHX resistance. Regarding cross-resistance, increased resistance to various antibiotic classes has been displayed experimentally by both Gram-positive (e.g., *Enterococcus faecium* and *Enterococcus faecalis*) and Gram-negative (e.g., *Enterobacter cloacae* or *Escherichia coli*) bacteria when exposed to sub-lethal concentrations of CHX (106, 107). While it is difficult to determine the clinical impact of these results and make a definitive conclusion about the increasing prevalence of CHX resistance, these findings stress the importance of using an adequate concentration of antiseptic in appropriate cases and support the argument for using antiseptics with a low selection pressure for resistance, such as alcohol and PI (106–108).

## 7 Conclusion

Antiseptics are an integral part of the preoperative preparation of surgical patients. Appropriate selection and use is necessary in order to prevent the occurrence of surgical site infections, adverse effects, and potential for increasing resistance to antimicrobials. At this time, the authors recognize that research does not support a single best antiseptic, however, the authors prefer a commercially available CHX-containing alcohol-based skin preparation when not contraindicated. Future choice of antiseptics would need to take into account resistance, especially to CHX. Continued research is needed to help fill gaps in the current knowledge such as optimal preparation procedures for the various surgical sites, and the clinical impact of decreased susceptibility to CHX and other antiseptics.

## Author contributions

KT: Conceptualization, Supervision, Writing – original draft, Writing – review & editing. AN: Conceptualization, Data curation, Writing – original draft, Writing – review & editing.

## References

[1] Stickney DNG Mankin KMT. The impact of postdischarge surveillance on surgical site infection diagnosis. Vet Surg. (2018) 47:66–73. doi: 10.1111/vsu.12738, PMID: 29094371

[2] Turk R Singh A Weese JS. Prospective surgical site infection surveillance in dogs. Vet Surg. (2015) 44:2–8. doi: 10.1111/j.1532-950x.2014.12267.x, PMID: 25196800

[3] Eugster S Schawalder P Gaschen F Boerlin P. A prospective study of postoperative surgical site infections in dogs and cats. Vet Surg. (2004) 33:542–50. doi: 10.1111/j.1532-950x.2004.04076.x, PMID: 15362994

[4] Mankin KMT Cohen ND. Randomized, controlled clinical trial to assess the effect of antimicrobial-impregnated suture on the incidence of surgical site infections in dogs and cats. J Am Vet Med Assoc. (2020) 257:62–9. doi: 10.2460/javma.257.1.62 32538702

[5] Mankin KMT Jeffery ND Kerwin SC. The impact of a surgical checklist on surgical outcomes in an academic institution. Vet Surg. (2021) 50:848–57. doi: 10.1111/vsu.13629, PMID: 33797097

[6] Mayhew PD Freeman L Kwan T Brown DC. Comparison of surgical site infection rates in clean and clean-contaminated wounds in dogs and cats after minimally invasive versus open surgery: 179 cases (2007–2008). J Am Vet Med Assoc. (2012) 240:193–8. doi: 10.2460/javma.240.2.193, PMID: 22217028

[7] Corr SA Brown C. A comparison of outcomes following tibial plateau levelling osteotomy and cranial tibial wedge osteotomy procedures. Vet Comp Orthopaed. (2007) 20:312–9. doi: 10.1160/vcot-07-02-0013, PMID: 18038011

[8] Sebben JE. Surgical antiseptics. J Am Acad Dermatol. (1983) 9:759–65. doi: 10.1016/S0190-9622(83)70192-1 6643773

[9] Nakayama DK. Antisepsis and asepsis and how they shaped modern surgery. Am Surg. (2018) 84:766–71. doi: 10.1177/000313481808400616, PMID: 29981599

[10] Boothe HW. Antiseptics and disinfectants. Vet Clin North Am Small Anim Pract. (1998) 28:233–48. doi: 10.1016/s0195-5616(98)82003-2 9556847

[11] McDonnell G Russell AD. Antiseptics and disinfectants: activity, action, and resistance. Clin Microbiol Rev. (1999) 12:147–79. doi: 10.1128/cmr.12.1.147, PMID: 9880479 PMC88911

[12] Gilbert P Moore LE. Cationic antiseptics: diversity of action under a common epithet. J Appl Microbiol. (2005) 99:703–15. doi: 10.1111/j.1365-2672.2005.02664.x, PMID: 16162221

[13] Horner C Mawer D Wilcox M. Reduced susceptibility to chlorhexidine in staphylococci: is it increasing and does it matter? J Antimicrob Chemoth. (2012) 67:2547–59. doi: 10.1093/jac/dks284, PMID: 22833635

[14] Bartzokas CA Corkill JE Makin T. Evaluation of the skin disinfecting activity and cumulative effect of chlorhexidine and Triclosan Handwash preparations on hands artificially contaminated with *Serratia marcescens*. Infect Control. (1987) 8:163–7. doi: 10.1017/s0195941700065838, PMID: 3294700

[15] Hibbard JS. Analyses comparing the antimicrobial activity and safety of current antiseptic agents: a review. J Infus Nurs. (2005) 28:194–207. doi: 10.1097/00129804-200505000-00008, PMID: 15912075

[16] Rutter JD Angiulo K Macinga DR. Measuring residual activity of topical antimicrobials: is the residual activity of chlorhexidine an artefact of laboratory methods? J Hosp Infect. (2014) 88:113–5. doi: 10.1016/j.jhin.2014.06.010, PMID: 25078726

[17] Kaiser N Klein D Karanja P Greten Z Newman J. Inactivation of chlorhexidine gluconate on skin by incompatible alcohol hand sanitizing gels. Am J Infect Control. (2009) 37:569–73. doi: 10.1016/j.ajic.2008.12.008, PMID: 19398245

[18] Benson L LeBlanc D Bush L White J. The effects of surfactant systems and moisturizing products on the residual activity of a chlorhexidine gluconate Handwash using a pigskin substrate. Infect Control Hosp Epidemiol. (1990) 11:67–70. doi: 10.2307/30144264, PMID: 2179400

[19] Walsh B Drabu PHBYJ Drabu YJ. The effect of handcream on the antibacterial activity of chlorhexidine gluconate. J Hosp Infect. (1985) 9:30–3. doi: 10.1016/0195-6701(87)90091-0 2880895

[20] Evans LKM Knowles TG Werrett G Holt PE. The efficacy of chlorhexidine gluconate in canine skin preparation – practice survey and clinical trials. J Small Anim Pract. (2009) 50:458–65. doi: 10.1111/j.1748-5827.2009.00773.x, PMID: 19769666

[21] Aly R. Antimicrobial activity of chlorhexidine gluconate against natural and artificial contamination during simulation of in-use conditions. J Pharm Sci. (1981) 70:964–4. doi: 10.1002/jps.2600700839, PMID: 7310676

[22] Oie S Kamiya A. Microbial contamination of antiseptics and disinfectants. Am J Infect Control. (1996) 24:389–95. doi: 10.1016/s0196-6553(96)90027-9 8902114

[23] Davids BI Davidson MJ TenBroeck SH Colahan PT Oli MW. Efficacy of mechanical versus non-mechanical sterile preoperative skin preparation with chlorhexidine gluconate 4% solution. Vet Surg. (2015) 44:648–52. doi: 10.1111/vsu.12335, PMID: 25913145

[24] Swales N Cogan T. Failure to achieve asepsis following surgical skin preparation is influenced by bacterial resistance to chlorhexidine, but not skin preparation technique. Vet Nurs J. (2017) 32:224–7. doi: 10.1080/17415349.2017.1328994

[25] Osuna DJ DeYoung DJ Walker RL. Comparison of three skin preparation techniques part 2: clinical trial in 100 dogs. Vet Surg. (1990) 19:20–3. doi: 10.1111/j.1532-950x.1990.tb01137.x, PMID: 2405580

[26] Berríos-Torres SI Umscheid CA Bratzler DW Leas B Stone EC Kelz RR. Centers for Disease Control and Prevention guideline for the prevention of surgical site infection, 2017. JAMA Surg. (2017) 152:784–91. doi: 10.1001/jamasurg.2017.0904, PMID: 28467526

[27] Webster J Osborne S. Preoperative bathing or showering with skin antiseptics to prevent surgical site infection. Cochrane Database Syst Rev. (2015) 2015:CD004985. doi: 10.1002/14651858.cd004985.pub5, PMID: 25927093 PMC10120916

[28] De C FLM Cota GF Pinto TS Ercole FF. Preoperative bathing of the surgical site with chlorhexidine for infection prevention: systematic review with meta-analysis. Am J Infect Control. (2017) 45:343–9. doi: 10.1016/j.ajic.2016.12.003, PMID: 28109628

[29] Kapadia BH Zhou PL Jauregui JJ Mont MA. Does preadmission cutaneous chlorhexidine preparation reduce surgical site infections after Total knee arthroplasty&quest. Clin Orthop Relat Res. (2016) 474:1592–8. doi: 10.1007/s11999-016-4767-6, PMID: 26956247 PMC4887366

[30] Kapadia BH Elmallah RK Mont MA. A randomized, clinical trial of preadmission chlorhexidine skin preparation for lower extremity Total joint arthroplasty. J Arthroplast. (2016) 31:2856–61. doi: 10.1016/j.arth.2016.05.043, PMID: 27365294

[31] Edmiston CE Lee CJ Krepel CJ Spencer M Leaper D Brown KR. Evidence for a standardized preadmission showering regimen to achieve maximal antiseptic skin surface concentrations of chlorhexidine gluconate, 4%, in surgical patients. JAMA Surg. (2015) 150:1027–33. doi: 10.1001/jamasurg.2015.2210, PMID: 26308490

[32] Coskun Ö Viskjer S. Chlorhexidine shampooing of dogs the night before elective surgery: are human recommendations applicable to veterinary medicine? Can J Vet Res. (2022) 86:306–10. 36211216 PMC9536351

[33] Sanchez IR Nusbaum KE Swaim SF Hale AS HendersonN RA McGuire JA. Chlorhexidine diacetate and povidone-iodine cytotoxicity to canine embryonic fibroblasts and *Staphylococcus aureus*. Vet Surg. (1988) 17:182–5. doi: 10.1111/j.1532-950x.1988.tb00995.x, PMID: 3238890

[34] Lozier S Pope E Berg J. Effects of four preparations of 0.05% chlorhexidine diacetate wound healing in dogs. Vet Surg. (1992) 21:107–12. doi: 10.1111/j.1532-950X.1992.tb00025.x, PMID: 1626379

[35] Sanchez IR Swaim SF Nsubaum KE Hale AS Henderson RA McGuire JA. Effects of chlorhexidine diacetate and povidone-lodine on wound healing in dogs. Vet Surg. (1988) 17:291–5. doi: 10.1111/j.1532-950x.1988.tb01019.x, PMID: 3232321

[36] Hennessey TD. Some antibacterial properties of chlorhexidine. J Periodontal Res. (1973) 8:61–7. doi: 10.1111/j.1600-0765.1973.tb02166.x 4269602

[37] Gélinas P Goulet J. Neutralization of the activity of eight disinfectants by organic matter. J Appl Microbiol. (1983) 54:243–7. doi: 10.1111/j.1365-2672.1983.tb02613.x, PMID: 6687886

[38] Perez R Freeman S Sohmer H Sichel JY. Vestibular and Cochlear ototoxicity of topical antiseptics assessed by evoked potentials. Laryngoscope. (2000) 110:1522–7. doi: 10.1097/00005537-200009000-00021, PMID: 10983954

[39] Bicknell PG. Sensorineural deafness following myringoplasty operations. J Laryngol Otol. (1971) 85:957–62. doi: 10.1017/s0022215100074272 5571878

[40] Merchant SR Neer TM Tedford BL Twedt AC Cheramie PM Strain GM. Ototoxicity assessment of a chlorhexidine otic preparation in dogs. Prog Vet Neurol. (2015) 4:72–5.

[41] Harvey R. Use of topical ear cleaners in small animals. In Pract. (2006) 28:131–5. doi: 10.1136/inpract.28.3.131

[42] Igarashi Y Oka Y. Mucosal injuries following intratympanic applications of chlorhexidine gluconate in the cat. Arch Otorhinolaryngol. (1988) 245:273–8. doi: 10.1007/bf00464629, PMID: 3245798

[43] Anderson GM. Soft tissues of the oral cavity In: Tobias K Johnston S, editors. Veterinary surgery: Small animal, 2. 2nd Edn. (2018). 1641.

[44] Steinsapir KD Woodward JA. Chlorhexidine keratitis. Dermatologic Surg. (2017) 43:1–6. doi: 10.1097/dss.0000000000000822, PMID: 27399954

[45] Rae SMM Brown B Edelhauser HF. The corneal toxicity of presurgical skin antiseptics. Am J Ophthalmol. (1984) 97:221–32. doi: 10.1016/s0002-9394(14)76094-5, PMID: 6696033

[46] Morgan JP Haug RH Kosman JW. Antimicrobial skin preparations for the maxillofacial region. J Oral Maxil Surg. (1996) 54:89–94. doi: 10.1016/s0278-2391(96)90312-2, PMID: 8531005

[47] Fowler JD Schuh JCL. Preoperative chemical preparation of the eye: a comparison of chlorhexidine diacetate, chlorhexidine gluconate, and povidone-lodine. J Am Vet Med Assoc. (1992) 28:451–7.

[48] Gili NJ Noren T Törnquist E Crafoord S Bäckman A. Preoperative preparation of eye with chlorhexidine solution significantly reduces bacterial load prior to 23-gauge vitrectomy in Swedish health care. BMC Ophthalmol. (2018) 18:167. doi: 10.1186/s12886-018-0844-9, PMID: 29996791 PMC6042411

[49] Greener Y McCartney M Jordan L Schmitt D Youkilis EJ. Assessment of the systemic effects, primary dermal irritation, and ocular irritation of chlorhexidine acetate solutions. Int J Toxicol. (1985) 4:309–19. doi: 10.3109/10915818509078694

[50] Harper WES Matz LR. The effect of chlorhexidine irrigation of the bladder in the rat. Brit J Urol. (1975) 47:539–43. doi: 10.1111/j.1464-410x.1975.tb06257.x, PMID: 1191923

[51] Wilson DG Cooley AJ Macwilliams PS Markel MD. Effects of 0.05% chlorhexidine lavage on the Tarsocrural joints of horses. Vet Surg. (1994) 23:442–7. doi: 10.1111/j.1532-950X.1994.tb00505.x, PMID: 7871707

[52] Case DE McAinsh J Rushton A Winrow MJ. (1976)“Chlorhexidine: attempts to detect percutaneous absorption in man” in Special Problems in Chemotherapy. eds. Williams JD. Geddes AM. Chemotherapy, vol 3. (Boston, MA: Springer).

[53] Silvestri DL McEnery-Stonelake M. Chlorhexidine. Dermatitis. (2013) 24:112–8. doi: 10.1097/der.0b013e3182905561 23665831

[54] Heinemann C Sinaiko R Maibach HI. Immunological contact Urticaria and anaphylaxis to chlorhexidine: overview. Curr Probl Dermatol. (2002) 1:186–94. doi: 10.1159/000066145

[55] Zamora JL. Chemical and microbiologic characteristics and toxicity of povidone-iodine solutions. Am J Surg. (1986) 151:400–6. doi: 10.1016/0002-9610(86)90477-0, PMID: 3513654

[56] Durani P Leaper D. Povidone–iodine: use in hand disinfection, skin preparation and antiseptic irrigation. Int Wound J. (2008) 5:376–87. doi: 10.1111/j.1742-481x.2007.00405.x, PMID: 18593388 PMC7951395

[57] Bigliardi PL Alsagoff SAL El-Kafrawi HY Pyon JK Wa CTC Villa MA. Povidone iodine in wound healing: a review of current concepts and practices. Int J Surg. (2017) 44:260–8. doi: 10.1016/j.ijsu.2017.06.073, PMID: 28648795

[58] Rutala WA. APIC guideline for selection and use of disinfectants. Am J Infect Control. (1996) 24:313–42. doi: 10.1016/s0196-6553(96)90066-8, PMID: 8870916

[59] Larson EL Committee 1992 1993, and 1994 APIC Guidelines. APIC guidelines for handwashing and hand antisepsis in health care settings. Am J Infect Control. (1995) 23:251–69. doi: 10.1016/0196-6553(95)90070-5, PMID: 7503437

[60] Atemnkeng MA Plaizier-Vercammen J Schuermans A. Comparison of free and bound iodine and iodide species as a function of the dilution of three commercial povidone–iodine formulations and their microbicidal activity. Int J Pharm. (2006) 317:161–6. doi: 10.1016/j.ijpharm.2006.03.013, PMID: 16650702

[61] Berkelman RL Holland BW Anderson RL. Increased bactericidal activity of dilute preparations of povidone-iodine solutions. J Clin Microbiol. (1982) 15:635–9. doi: 10.1128/jcm.15.4.635-639.1982, PMID: 7040461 PMC272159

[62] Zamora JL Price MF Chuang P Gentry LO. Inhibition of povidone-iodine’s bactericidal activity by common organic substances: an experimental study. Surgery. (1985) 98:25–9. PMID: 4012604

[63] Ghogawala Z Furtado D. *In vitro* and *in vivo* bactericidal activities of 10, 2.5, and 1% povidone-iodine solution. Am J Hosp Pharm. (1990) 47:1562–6. PMID: 2368748

[64] Hemani ML Lepor H. Skin preparation for the prevention of surgical site infection: which agent is best? Rev Urol. (2009) 11:190–5. doi: 10.3909/riu0467 20111631 PMC2809986

[65] Schmidt AL Oliver SP Fydenkevez ME. Germicidal persistence of teat dips by modified excised teat Procedure1. J Dairy Sci. (1985) 68:158–62. doi: 10.3168/jds.s0022-0302(85)80810-9 3884679

[66] Berkelman RL Anderson RL Davis BJ Highsmith AK Petersen NJ Bond WW. Intrinsic bacterial contamination of a commercial lodophor solution: investigation of the implicated manufacturing plant. App Environ Microbiol. (1984) 47:752–6. doi: 10.1128/aem.47.4.752-756.1984, PMID: 16346513 PMC239760

[67] O’Rourke E Runyan D O’Leary J Stern J. Contaminated iodophor in the operating room. Am J Infect Control. (2003) 31:255–6. doi: 10.1067/mic.2003.13, PMID: 12806364

[68] Goswami K Austin MS. Intraoperative povidone-iodine irrigation for infection prevention. Arthroplast Today. (2019) 5:306–8. doi: 10.1016/j.artd.2019.04.004, PMID: 31516971 PMC6728529

[69] Israel SK Jaramillo E Liska WD. Preclosure povidone-iodine lavage in total hip replacement surgery: infection outcomes and cost–benefit analysis. Vet Surg. (2023) 52:33–41. doi: 10.1111/vsu.13910, PMID: 36411945

[70] Roberts SM Severin GA Lavach JD. Antibacterial activity of dilute povidone-iodine solutions used for ocular surface disinfection in dogs. Am J Vet Res. (1986) 47:1207–10. PMID: 3729119

[71] Ferguson AW Scott JA McGavigan J Elton RA McLean J Schmidt U. Comparison of 5% povidone-iodine solution against 1% povidone-iodine solution in preoperative cataract surgery antisepsis: a prospective randomised double blind study. Brit J Ophthalmol. (2003) 87:163–7. doi: 10.1136/bjo.87.2.163, PMID: 12543744 PMC1771501

[72] Birnbach DJ Meadows W Stein DJ Murray O Thys DM Sordillo EM. Comparison of povidone iodine and DuraPrep, an Iodophor-in-isopropyl alcohol solution, for skin disinfection prior to epidural catheter insertion in Parturients. Anesthesiology. (2003) 98:164–9. doi: 10.1097/00000542-200301000-00026, PMID: 12502993

[73] Osuna DJ DeYoung DJ Walker RL. Comparison of an antimicrobial adhesive drape and povidone-lodine preoperative skin preparation in dogs. Vet Surg. (1992) 21:458–62. doi: 10.1111/j.1532-950x.1992.tb00081.x, PMID: 1455649

[74] Owen LJ Gines JA Knowles TG Holt PE. Efficacy of adhesive incise drapes in preventing bacterial contamination of clean canine surgical wounds. Vet Surg. (2009) 38:732–7. doi: 10.1111/j.1532-950x.2009.00537.x, PMID: 19674416

[75] Webster J Alghamdi A. Use of plastic adhesive drapes during surgery for preventing surgical site infection. Cochrane Database Syst Rev. (2015) 2019:CD006353. doi: 10.1002/14651858.cd006353.pub4, PMID: 25901509 PMC6575154

[76] Osuna DJ DeYoung DJ Walker RL. Comparison of three skin preparation techniques in the dog part 1: experimental trial. Vet Surg. (1990) 19:14–9. doi: 10.1111/j.1532-950x.1990.tb01136.x, PMID: 2301156

[77] Neihaus SA Hathcock TL Boothe DM Goring RL. Presurgical antiseptic efficacy of chlorhexidine diacetate and povidone-iodine in the canine preputial cavity. J Am Anim Hosp Assoc. (2011) 47:406–12. doi: 10.5326/jaaha-ms-5681, PMID: 22058347

[78] Cruz FD Brown DH Leikin JB Franklin C Hryhorczuk DO. Iodine absorption after topical administration. West J Med. (1987) 146:43–5. 3825108 PMC1307179

[79] Adams D Quayum M Worthington T Lambert P Elliott T. Evaluation of a 2% chlorhexidine gluconate in 70% isopropyl alcohol skin disinfectant. J Hosp Infect. (2005) 61:287–90. doi: 10.1016/j.jhin.2005.05.015 16221509

[80] Lowbury EJL Lilly HA Ayliffe GAJ. Preoperative disinfection of surgeons’ hands: use of alcoholic solutions and effects of gloves on skin Flora. Br Med J. (2016) 4:369–72. doi: 10.1136/bmj.4.5941.369 PMC1612461 4609555

[81] Larson E Bobo L. Effective hand degerming in the presence of blood. J Emerg Med. (1992) 10:7–11. doi: 10.1016/0736-4679(92)90003-c, PMID: 1629595

[82] Isaza D DiGangi BA Isaza N Isaza R. Impact of surgical preparatory rinses with isopropyl alcohol or water on perioperative body temperature in pediatric female dogs and cats. Vet Anaesth Analg. (2021) 48:198–204. doi: 10.1016/j.vaa.2020.11.006, PMID: 33589395

[83] Martinez TT Jaeger RW deCastro FJ Thompson MW Hamilton MF. A comparison of the absorption and metabolism of isopropyl alcohol by oral, dermal and inhalation routes. Vet Hum Toxicol. (1986) 28:233–6. PMID: 3727356

[84] Garrison RF. Acute poisoning from use of Iso-propyl alcohol in tepid sponging. J Am Vet Med Assoc. (1953) 152:317–8. doi: 10.1001/jama.1953.63690040001007, PMID: 13044524

[85] Grant DH. The antiseptic and bactericidal properties of isopropyl alcohol. Am J Med Sci. (1923) 166:261–4. doi: 10.1097/00000441-192308000-00014

[86] Lee I Agarwal RK Lee BY Fishman NO Umscheid CA. Systematic review and cost analysis comparing use of chlorhexidine with use of iodine for preoperative skin antisepsis to prevent surgical site infection. Infect Control Hosp Epidemiol. (2010) 31:1219–29. doi: 10.1086/657134, PMID: 20969449 PMC3833867

[87] Noorani A Rabey N Walsh SR Davies RJ. Systematic review and meta-analysis of preoperative antisepsis with chlorhexidine versus povidone–iodine in clean-contaminated surgery. Br J Surg. (2010) 97:1614–20. doi: 10.1002/bjs.7214, PMID: 20878942

[88] Chen S Chen JW Guo B Xu CC. Preoperative antisepsis with chlorhexidine versus povidone-iodine for the prevention of surgical site infection: a systematic review and Meta-analysis. World J Surg. (2020) 44:1412–24. doi: 10.1007/s00268-020-05384-7, PMID: 31996985

[89] Privitera GP Costa AL Brusaferro S Chirletti P Crosasso P Massimetti G. Skin antisepsis with chlorhexidine versus iodine for the prevention of surgical site infection: a systematic review and meta-analysis. Am J Infect Control. (2017) 45:180–9. doi: 10.1016/j.ajic.2016.09.017, PMID: 27838164

[90] Kamel C McGahan L Polisena J Mierzwinski-Urban M Embil JM. Preoperative skin antiseptic preparations for preventing surgical site infections: a systematic review. Infect Control Hosp Epidemiol. (2012) 33:608–17. doi: 10.1086/665723 22561717

[91] Dumville JC McFarlane E Edwards P Lipp A Holmes A Liu Z. Preoperative skin antiseptics for preventing surgical wound infections after clean surgery. Cochrane Database Syst Rev. (2013) 3:CD003949. doi: 10.1002/14651858.cd003949.pub3, PMID: 23543526

[92] Marchionatti E Constant C Steiner A. Preoperative skin asepsis protocols using chlorhexidine versus povidone-iodine in veterinary surgery: a systematic review and meta-analysis. Vet Surg. (2022) 51:744–52. doi: 10.1111/vsu.13810, PMID: 35437786 PMC9321991

[93] Amber EI Henderson RA Swaim SF Gray BW. A comparison of antimicrobial efficacy and tissue reaction of four antiseptics on canine wounds. Vet Surg. (1983) 12:63–8. doi: 10.1111/j.1532-950x.1983.tb00708.x

[94] Nye AK Rogovskyy A Lazarus MA Amore R Mankin KMT. Effectiveness of chlorhexidine diacetate and povidone-iodine in antiseptic preparation of the canine external ear canal prior to total ear canal ablation with bulla osteotomy procedure: a preliminary study. Vet Med Sci. (2023) 9:1998–2005. doi: 10.1002/vms3.1200, PMID: 37418348 PMC10508515

[95] Phillips MF Vasseur PB Gregory CR. Chlorhexidine iodine for preoperative preparation of the skin: a prospective randomized comparison in dogs and cats. J Am Anim Hosp Assoc. (1991) 27:105–8.

[96] Belo L Serrano I Cunha E Carneiro C Tavares L Carreira LM. Skin asepsis protocols as a preventive measure of surgical site infections in dogs: chlorhexidine–alcohol versus povidone–iodine. BMC Vet Res. (2018) 14:95. doi: 10.1186/s12917-018-1368-5, PMID: 29540169 PMC5852956

[97] Gibson KL Donald AW Hariharan H McCarville C. Comparison of two pre-surgical skin preparation techniques. Can J Vet Res Revue Can De Recherche Vétérinaire. (1997) 61:154–6. PMC1189392 9114967

[98] Swaim SF Riddell KP Geiger DL Hathcock TL McGuire JA. Evaluation of surgical scrub and antiseptic solutions for surgical preparation of canine paws. J Am Vet Med Assoc. (1991) 198:1941–5. doi: 10.2460/javma.1991.198.11.1941, PMID: 1874671

[99] Maiwald M Chan ESY. The forgotten role of alcohol: a systematic review and Meta-analysis of the clinical efficacy and perceived role of chlorhexidine in skin antisepsis. PLoS One. (2012) 7:e44277. doi: 10.1371/journal.pone.0044277, PMID: 22984485 PMC3434203

[100] Kaoutzanis C Kavanagh CM Leichtle SW Welch KB Talsma A Vandewarker JF. Chlorhexidine with isopropyl alcohol versus iodine Povacrylex with isopropyl alcohol and alcohol-versus nonalcohol-based skin preparations. Dis Colon Rectum. (2015) 58:588–96. doi: 10.1097/dcr.0000000000000379, PMID: 25944431

[101] Asimus E Palierne S Blondel M Pollet V Ferran A Bousquet-Melou A. Comparison of hydroalcoholic rubbing and conventional chlorhexidine scrubbing for aseptic skin preparation in dogs. Vet Surg. (2019) 48:1466–72. doi: 10.1111/vsu.13222, PMID: 31034647

[102] Peel TN Watson E Lee SJ. Randomised controlled trials of alcohol-based surgical site skin preparation for the prevention of surgical site infections: systematic review and Meta-analysis. J Clin Med. (2021) 10:663. doi: 10.3390/jcm10040663, PMID: 33572218 PMC7914441

[103] SCENIHR (Scientific Committee on Emerging and Newly Identified Health Risks), Assessment of the antibiotic resistance effects of biocides. (2009). Available at: https://ec.europa.eu/health/ph_risk/committees/04_scenihr/docs/scenihr_o_021.pdf

[104] Boothe DM Boothe HW. Antimicrobial considerations in the perioperative patient. Vet Clin North Am Small Anim Pract. (2015) 45:585–608. doi: 10.1016/j.cvsm.2015.01.006 25758849

[105] Russell AD. Do biocides select for antibiotic resistance?*. J Pharm Pharmacol. (2000) 52:227–33. doi: 10.1211/0022357001773742 10714955

[106] Kampf G. Antibiotic resistance can be enhanced in gram-positive species by some biocidal agents used for disinfection. Antibiotics. (2019) 8:13. doi: 10.3390/antibiotics8010013, PMID: 30717270 PMC6466599

[107] Kampf G. Biocidal agents used for disinfection can enhance antibiotic resistance in gram-negative species. Antibiotics. (2018) 7:110. doi: 10.3390/antibiotics7040110, PMID: 30558235 PMC6316403

[108] Kampf G. Acquired resistance to chlorhexidine – is it time to establish an ‘antiseptic stewardship’ initiative? J Hosp Infect. (2016) 94:213–27. doi: 10.1016/j.jhin.2016.08.018, PMID: 27671220

